# Heterogeneity of Plaque Structural Stress Is Increased in Plaques Leading to MACE: Insights From the PROSPECT Study

**DOI:** 10.1016/j.jcmg.2019.05.024

**Published:** 2020-05

**Authors:** Charis Costopoulos, Akiko Maehara, Yuan Huang, Adam J. Brown, Jonathan H. Gillard, Zhongzhao Teng, Gregg W. Stone, Martin R. Bennett

**Affiliations:** a Division of Cardiovascular Medicine, University of Cambridge, Cambridge, United Kingdom; b Cardiovascular Research Foundation, New York City, New York; c Department of Engineering and Physical Sciences Research Council, Centre for Mathematical and Statistical Analysis of Multimodal Imaging, University of Cambridge, Cambridge, United Kingdom; d Department of Radiology, University of Cambridge, Cambridge, United Kingdom; e Department of Engineering, University of Cambridge, Cambridge, United Kingdom

**Keywords:** intravascular imaging, myocardial infarction, plaque structural stress, thin-cap fibroatheroma, FEA, finite element analysis, HI, heterogeneity index, MACE, major adverse cardiovascular event(s), MLA, minimal luminal area, PB, plaque burden, PSS, plaque structural stress, VH-IVUS, virtual histology intravascular ultrasonography, VH-TCFA, virtual histology thin-cap fibroatheroma

## Abstract

**Objectives:**

This study sought to determine if plaque structural stress (PSS) and other plaque stress parameters are increased in plaques that cause future major adverse cardiovascular event(s) (MACE) and if incorporating these parameters improves predictive capability of intravascular ultrasonography (IVUS).

**Background:**

Less than 10% of coronary plaques identified as high-risk by intravascular imaging result in subsequent MACE. Thus, more specific measurements of plaque vulnerability are required for effective risk stratification.

**Methods:**

Propensity score matching in the PROSPECT (Providing Regional Observations to Study Predictors of Events in the Coronary Tree) study plaque cohort resulted in 35 nonculprit lesions (NCL) associated with future MACE and 66 matched NCL that remained clinically silent. PSS was calculated by finite element analysis as the mechanical loading within the plaque structure in the periluminal region.

**Results:**

PSS was increased in the minimal luminal area (MLA) regions of NCL MACE versus no MACE plaques for all plaques (PSS: 112.1 ± 5.5 kPa vs. 90.4 ± 3.3 kPa, respectively; p = 0.001) and virtual histology thin-cap fibroatheromas (VH-TCFAs) (PSS: 119.2 ± 6.6 kPa vs. 95.8 ± 5.0 kPa, respectively; p = 0.005). However, PSS was heterogeneous over short segments, and PSS heterogeneity index (HI) was markedly greater in NCL MACE than in no-MACE VH-TCFAs (HI: 0.43 ± 0.05 vs. 0.29 ± 0.03, respectively; p = 0.01). Inclusion of PSS in plaque assessment improved the identification of NCLs that led to MACE, including in VH-TCFAs (p = 0.03) and plaques with MLA ≤4 mm^2^ (p = 0.03). Incorporation of an HI further improved the ability of PSS to identify MACE NCLs in a variety of plaque subtypes including VH-TCFA (p = 0.001) and plaques with MLA ≤4 mm^2^ (p = 0.002).

**Conclusions:**

PSS and variations in PSS are increased in the peri-MLA regions of plaques that lead to MACE. Moreover, longitudinal heterogeneity in PSS is markedly increased in MACE plaques, especially VH-TCFAs, potentially predisposing to plaque rupture. Incorporation of PSS and heterogeneity in PSS may improve the ability of IVUS to predict MACE.

Cardiovascular disease is a leading cause of mortality worldwide (1), and most deaths are attributable to ischemic heart disease. Techniques that can identify coronary plaques at risk for adverse events are therefore of particular interest. Virtual histology intravascular ultrasonography (VH-IVUS) can determine both plaque size and composition, resulting in an imaging-based plaque classification. PROSPECT (Providing Regional Observations to Study Predictors of Events in the Coronary Tree), the largest VH-IVUS study to date, showed that minimal luminal area (MLA) ≤4.0 mm^2^, plaque burden (PB) ≥70% at the MLA, and the presence of virtual histology thin-cap fibroatheroma (VH-TCFA) were independent predictors of nonculprit (NCL) future major adverse cardiovascular event(s) (MACE) over a 3-year period (2). In contrast, NCL nonfibroatheromas were rarely associated with MACE over the same interval (3). Subsequently both the VIVA (VH-IVUS in vulnerable atherosclerosis) and the AtheroRemoIVUS (European Collaborative Project on Inflammation and Vascular Wall Remodeling in Atherosclerosis-Intravascular Ultrasound Study; NCT01789411) studies found that VH-TCFA and PB ≥70% were associated with future events 4, 5. Although the consistency of all 3 prospective studies validates these features to identify higher risk plaques, <10% of NCL VH-TCFAs led to MACE in all 3 studies, indicating that more specific techniques are required to better characterize coronary plaques.

Plaque structural stress (PSS) is the stress located inside an atherosclerotic plaque due to plaque structure and composition, and is affected by vessel expansion and stretch induced by arterial pressure. PSS is linked to plaque rupture both ex and in vivo 6, 7, so incorporation of PSS into coronary plaque assessment may improve the ability of imaging to identify high-risk coronary plaques. Indeed, PSS is increased in culprit plaques of patients presenting with acute coronary syndrome (ACS) versus stable angina (8) and in the peri-MLA region of those showing rupture versus no rupture (9). PSS was increased in NCL MACE plaques in the VIVA study compared to no-MACE plaques with similar gray-scale and VH-IVUS characteristics (10). However, for plaque stress measurements to be useful clinically they need to be validated in larger multicenter studies, and other stress-based parameters may be more predictive of MACE than PSS alone. The current study aimed to determine if a) PSS of plaques responsible for NCL MACE in PROSPECT are increased compared to a propensity-score-matched control population; b) if other stress-based parameters were more discriminatory; and (c) if incorporating different PSS parameters provides incremental prognostic information over IVUS imaging alone.

## Methods

### Patient recruitment

The protocol for the PROSPECT (Providing Regional Observations to Study Predictors of Events in the Coronary Tree) study and inclusion and exclusion criteria have already been described (2) (Supplemental Appendix). Briefly, 697 patients with ACS were recruited across 37 U.S. and European sites after undergoing successful percutaneous coronary intervention for all coronary lesions responsible for the index event and completion of any other planned interventions. A total of 623 patients underwent 3-vessel gray-scale and VH-IVUS assessment, and medication therapy after discharge was followed according to guideline standards. Clinical follow-up occurred at 30 days, 6 months, and annually for at least 2 years (median: 3.4 years).

### VH-IVUS image acquisition and analysis

VH-IVUS was performed in the left main stem and proximal 6 to 8 cm of the major epicardial vessels using a 20-MHz synthetic aperture array 3.2-F catheter (Eagle Eye, In-Vision Gold, Volcano, Rancho Cordova, California) with motorized catheter pull back (0.5 mm/s) after the administration of glycerin trinitrate. A plaque was defined as ≥3 consecutive frames with PB ≥40% and classified as VH-TCFA, thick-cap fibroatheroma (VH-ThCFA), pathological intimal thickening (VH-PIT), fibrotic (VH-FT), or fibrocalcific plaque (VH-FCa). The MLA was defined as the IVUS frame with the smallest luminal area over the whole plaque. A lesion was classified as having PB ≥70% if PB at the MLA site was ≥70%. Analysis was performed off-line and was not used for procedural guidance.

### Clinical endpoints and definitions

Independent study monitors verified all data for case report forms. The pre-specified primary endpoint was the incidence of MACE defined as the composite of cardiac death, cardiac arrest, myocardial infarction, or hospitalization due to unstable or progressive angina according to Braunwald unstable angina classification and the Canadian Cardiovascular Society angina classification. The primary endpoint was adjudicated by a clinical events committee that had no knowledge of other patient data. Clinical events were attributed to culprit lesions or non-culprit lesions (NCL) based on follow-up angiography. If angiography was not performed, the event was classified as indeterminate.

### Biomechanical analysis

Vessel geometry and plaque composition were extracted from radiofrequency IVUS data and imported into dedicated analysis software (proprietary code, MATLAB R2012b, MathWorks, Inc, Natick, Massachusetts), allowing construction of 8,182 VH-IVUS models. Briefly, each VH-IVUS frame was segmented into its individual components using an in-house MATLAB code with the resulting segmented model undergoing dynamic 2D FEA simulations as described previously (Supplemental Appendix). A 65-μm layer of fibrous tissue was introduced during mesh generation to account for the limited axial resolution of VH-IVUS to detect a fibrous cap between lumen and necrotic core/dense calcium. Maximum principal stress was used to indicate the critical mechanical conditions, the PSS, with variations in PSS being the difference between PSS in systole and that in diastole. As plaque destabilization is a focal event, PSS across the whole plaque may not reflect PSS where plaque disruption occurs. Therefore, PSS and variations in PSS in the peri-MLA segments (≈4 mm proximal and distal to the MLA with PB ≥40%) were compared, as this represents extensive disease and where plaque disruption often occurs (9). The heterogeneity of PSS along the length of each plaque was also examined as this can amplify the effects of PSS or variations in PSS at areas of fibrous cap weakness thereby promoting plaque rupture. This was assessed by the heterogeneity index (HI), defined as the standard deviation of PSS divided by mean PSS or variations in PSS in the area of interest.

### Statistical analysis

**Figure 1:**
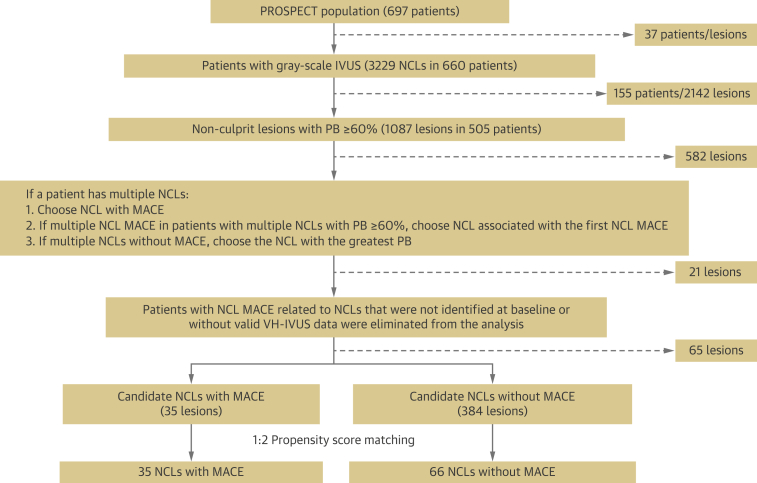
Patient and Plaque Populations Schematic representation of patient and plaque populations included in the study. MACE = major adverse cardiovascular event(s); NCL = nonculprit lesion; PB = plaque burden; VH-IVUS = virtual histology intravascular ultrasonography.

Propensity score matching with a 1:2 ratio was used to identify NCL MACE and no-MACE groups to minimize selection bias due to differences in patient and lesion characteristics. Only 1 lesion per patient was selected, and only those patients with a PB ≥60% at the MLA were included, because more than 85% of NCL MACE occurred in lesions with a PB ≥60%. In patients with multiple NCL, the lesion with the greatest PB was chosen. However, PSS was also calculated for all VH-IVUS frames with PB ≥40%. Both patient and lesion parameters were used in calculation of propensity scores. Patient-level parameters included presence of insulin-treated diabetes mellitus, history of percutaneous coronary intervention, age, and sex. Lesion-level parameters included PB at the MLA, MLA, and plaque classification (Supplemental Appendix). Propensity score matching generated 101 NCLs, 35 of which led to future MACE (Figure 1). The remaining 66 NCLs formed the control group. The C-statistic was 0.887, and the Hosmer-Lemeshow test p value was 0.931, confirming good discrimination and goodness-of-fit of the propensity score model. A total of 8,182 VH-IVUS frames were analyzed with a median of 66 (42 to 104) frames per plaque.

Data variables are median (quartile [Q]1 to Q3) and were compared using the Mann-Whitney *U* test. As each plaque had multiple VH-IVUS slices, a linear mixed-effects model was used to compare groups by using a random effect for plaque and fixed effects for group to account for clustering with results presented as mean ± SEM. All calculations were 2-tailed and a p value <0.05 was considered statistically significant. Receiver operating characteristic (ROC) curves were calculated by plotting sensitivity versus (1 − specificity), allowing calculation of the area under the curve (AUC) and the identification of PSS and heterogeneity in PSS cutoff thresholds that best predicted future MACE. Each cutoff value was subsequently applied to categorize PSS and heterogeneity in PSS into low and high groups, allowing generation of time-to-event curves that incorporated these. Time-to-event data are presented as Kaplan-Meier estimates of cumulative hazard and were compared using the log-rank method. Statistical analyses were performed using both SPSS version 19.0.0 software (IBM, Armonk, New York) and R version 2.10.1 software (R Foundation for Statistical Computing, Vienna, Austria).

## Results

### Baseline patient characteristics

A total of 101 patients were included in the analysis, 35 of whom experienced a NCL MACE. Patient demographics were similar between the 2 groups (Supplemental Table 1). There were no significant differences in rates of diabetes (28.6% vs. 19.7%, respectively; p = 0.31) or hypercholesterolemia (53.6% vs. 49.2%, respectively; p = 0.70). Initial clinical presentation was also similar between the MACE and no-MACE groups, with most patients presenting with non–ST-segment elevation myocardial infarction (68.6% vs. 72.7%, respectively; p = 0.66). Following treatment of culprit vessels, patients began medical therapy according to local guidelines. There were no significant differences between the 2 groups for antiplatelet, statin, angiotensin-converting enzyme inhibitor, or beta-blocker therapy, either at discharge or follow-up (Supplemental Table 2).

### Baseline nonculprit lesion angiographic and IVUS characteristics

Vessels of patients underwent quantitative angiographic coronary and IVUS analysis. There were no significant differences in the number of diseased vessels, defined as diameter of stenosis on quantitative angiographic coronary analysis >30%, or number of vessels with lesions (Supplemental Table 3). Gray-scale IVUS analysis revealed some differences between the 2 groups. Echolucent plaques were more frequent in the MACE group (34.3% vs. 12.1%, respectively; p = 0.008) but with similar NCL length (30.4 mm [range 19.5 to 41.4 mm] vs. 24.3 mm [range 15.7 to 36.7 mm], respectively; p = 0.11). There were no significant differences in other gray-scale IVUS characteristics (Supplemental Table 4). In both groups, virtual histology fibroatheroma was the predominant type of lesion (91.4% vs. 86.4%, respectively; p = 0.54), approximately 50% were VH-TCFA (57.1% vs. 47.0%, respectively; p = 0.33). There were no significant differences in overall plaque composition, defined as percent of fibrous tissue, fibrofatty tissue, necrotic core, or dense calcium, either over the whole plaque or at the MLA (Table 1).

**Table 1:** VH-IVUS Characteristics of MACE and No MACE Nonculprit Lesions

	MACE (n = 35)	No MACE (n = 66)	p Value
Lesion phenotype			
VH-TCFA	20/35 (57.1)	31/66 (47.0)	0.33
VH-ThCFA	12/35 (34.3)	26/66 (39.4)	0.61
VH-PIT	2/35 (5.7)	9/66 (13.6)	0.32
VH-FCa	1/35 (2.9)	0/66 (0.0)	0.35
VH-FT	0/35 (0.0)	0/66 (0.0)	NA
VH-TCFA or VH-ThCFA	32/35 (91.4)	57/66 (86.4)	0.54
Plaque data			
% NC volume	15.5 (9.0-21.5)	14.9 (8.8-23.1)	0.73
% DC volume	6.1 (2.7-9.8)	6.3 (3.6-9.4)	0.95
% FT volume	60.7 (54.5-65.9)	57.6 (53.7-62.0)	0.22
% FF volume	15.2 (10.9-22.5)	16.4 (10.2-23.2)	0.78
MLA site data			
% NC CSA	13.9 (8.7-25.1)	16.6 (8.9-29.1)	0.44
% DC CSA	5.0 (2.3-10.9)	6.0 (2.0-11.4)	0.73
% FT CSA	61.1 (53.7-68.8)	58.7 (49.0-64.2)	0.11
% FF CSA	11.1 (7.0-22.0)	12.6 (5.8-22.2)	0.80

Values are n/N (%) or median (interquartile range).

CSA = cross-sectional area; DC = dense calcium; FT = fibrous tissue; FF = fibrofatty; FCa = fibrocalcific; NC = necrotic core; MACE = major adverse cardiovascular event(s); MLA = minimum luminal area; PIT = pathological intimal thickening TCFA = thin-cap fibroatheroma; ThCFA = thick-cap fibroatheroma.

### PSS in nonculprit lesions with MACE at the PERI-MLA segments

**Figure 2:**
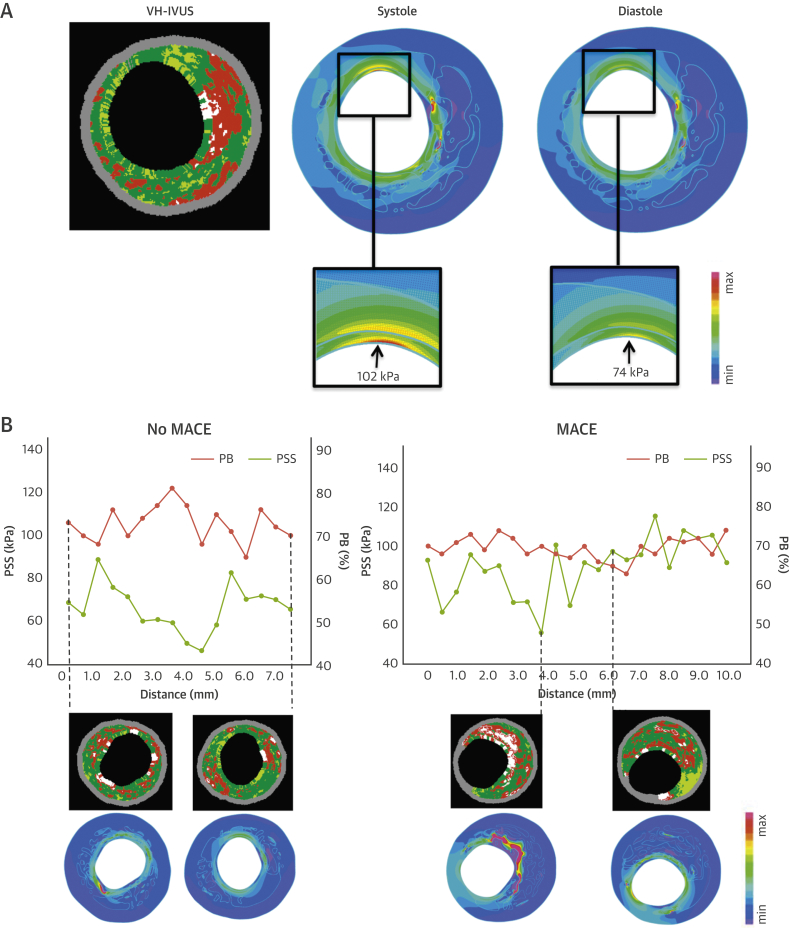
Temporal and Longitudinal Variation in PSS **(A)** VH-IVUS frame and PSS band plots in systole and in diastole. **(B)** Longitudinal variations of PSS in no-MACE and MACE plaques with examples of VH-IVUS images and corresponding PSS band plots. PSS = plaque structural stress; other abbreviations as in Figure 1.

A total of 101 nonculprit plaques (35 with MACE, 66 with no-MACE) were analyzed, generating 8,182 VH-IVUS frames, all of which underwent FEA to calculate mean PSS and PSS variations at systole and diastole (Figure 2A). PSS and variations in PSS across the entire plaque length were similar between the 2 groups, regardless of plaques subtype (Supplemental Figure 1). However, PSS was markedly heterogeneous across the whole plaque, even over short distances (Figure 2B), reflecting small changes in plaque composition at the luminal surface.

**Figure 3:**
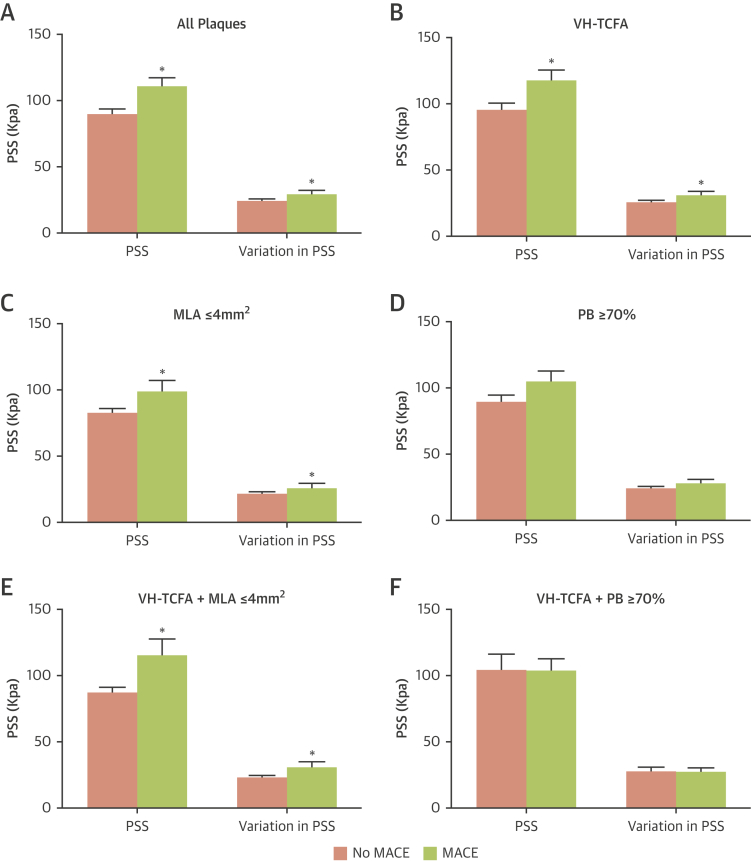
Comparison of PSS and Variations in PSS in Peri-MLA Segments With and Without Future MACE PSS and variations in PSS at **(A)** all plaques, **(B)** VH-TCFAs, **(C)** Plaques with MLA ≤4 mm^2^, **(D)** plaques with PB ≥70% at the MLA, **(E)** VH-TCFA + MLA ≤4mm^2^, and **(F)** VH-TCFA + PB ≥70%. p < 0.05. MLA = minimal luminal area; PB = plaque burden; PSS = plaque structural stress; VH-TCFA = virtual histology thin-cap fibroatheroma; other abbreviations as in Figure 1.

PSS and variations in PSS were increased in MACE compared to those in the no-MACE groups in the peri-MLA regions regardless of whether all plaques (PSS: 112.1 ± 5.5 kPa vs. 90.4 ± 3.3 kPa; p = 0.001; variations in PSS: 30.5 ± 1.5 kPa vs. 24.5 ± 0.9 kPa; p = 0.001) or only VH-TCFA plaques (PSS: 119.2 ± 6.6 kPa vs. 95.8 ± 5.0 kPa; p = 0.005; variations in PSS: 32.3 ± 1.8 kPa vs. 25.9 ± 1.4 kPa; p = 0.004) were examined (Figures 3A and 3B). PSS was also increased in MACE compared to those in no-MACE plaques with MLA ≤4 mm^2^ (PSS: 100.3 ± 7.5 kPa vs. 83.6 ± 2.7 kPa; p = 0.007; variations in PSS: 27.5 ± 2.1 kPa vs. 22.6 ± 0.7 kPa; p = 0.007) (Figure 3C) and with PB ≥70% at the MLA site, although this was of borderline statistical significance (106.4 ± 6.7 kPa vs. 90.1 ± 5.2 kPa; p = 0.05; variations in PSS: 29.1 ± 1.9 vs. 24.4 ± 1.4 kPa; p = 0.05) (Figure 3D). PSS and variations in PSS were also increased in the MACE group when VH-TCFAs with an MLA ≤4 mm^2^ specifically was examined (Figure 3E) but not VH-TCFA with PB ≥70% (Figure 3F).

Heterogeneity in PSS and variations in PSS was increased at the peri-MLA segment.

**Figure 4:**
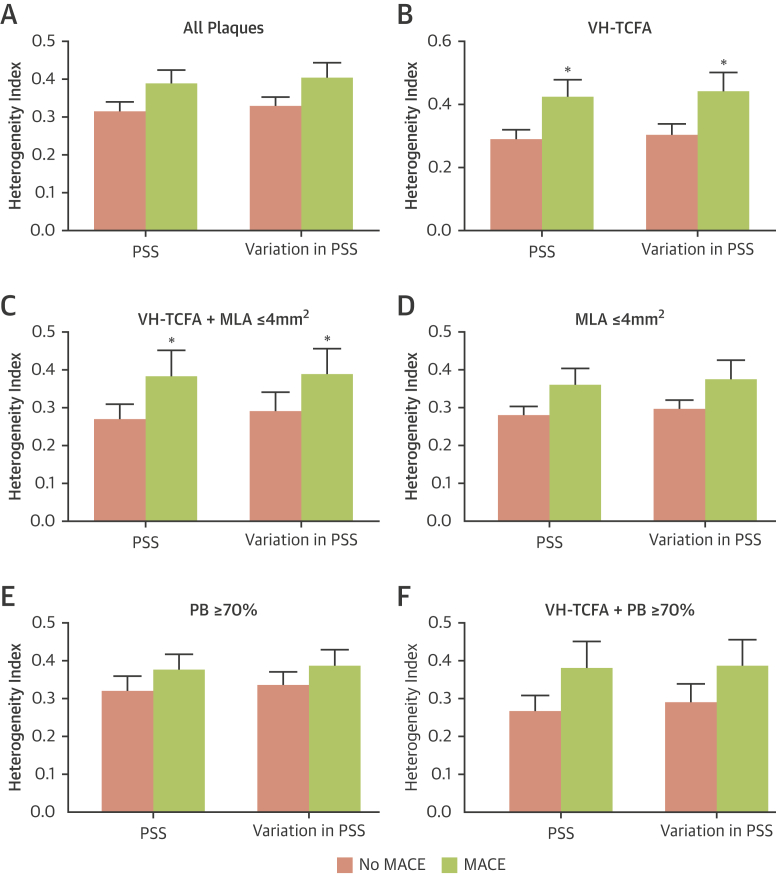
Comparison of PSS and Variation in PSS Heterogeneity in Peri-MLA Plaque Segments With and Without Future MACE Heterogeneity index of PSS or variations in PSS for **(A)** all plaques, **(B)** VH-TCFAs, **(C)** VH-TCFA + MLA ≤4 mm^2^, **(D)** plaques with MLA ≤4 mm^2^, **(E)** plaques with PB ≥ 70% at the MLA and **(F)** VH-TCFA + PB ≥70%. *p < 0.05. Abbreviations as in Figures 1 and 3.

Substantial differences were observed in PSS along the plaque length, even over short distances (Figure 2B, Supplemental Figure 2). Therefore the heterogeneity of PSS and variations in PSS in MACE versus no-MACE plaques were examined using a standard HI. Although heterogeneity of PSS or PSS variations in all plaques were similar between the 2 groups (PSS HI: p = 0.07; variations in PSS HI: p = 0.05) (Figure 4A), both parameters were markedly greater in MACE VH-TCFAs (PSS HI: p = 0.01; variations in PSS HI: p = 0.02) (Figure 4B), and in VH-TCFAs with MLA ≤4 mm^2^ (PSS HI: p = 0.02; variations in PSS HI: p = 0.03) (Figure 4C). Heterogeneity was similar in plaques with MLA ≤4 mm^2^ (Figure 4D), PB ≥70% at the MLA site (Figure 4E), or VH-TCFA plus PB ≥70% (Figure 4F) in both groups.

### Incorporation of PSS and heterogeneity in PSS improved prediction of MACE

In PROSPECT, VH-TCFAs (4.9% vs. 1.3%; p = 0.001), plaques with MLA ≤4 mm^2^ (5.3% vs. 1.1%; p = 0.001), and those with PB ≥70% at the MLA site (9.6% vs. 1.2%; p = 0.001) were more likely to lead to NCL MACE. However, 45.5% of NCL MACE plaques had an MLA >4 mm^2^, 54.5% had PB <70% at the MLA site, and 49.0% of MACE occurred in non-VH-ThCFA plaques (2) (Supplemental Figure 3). Therefore, PSS and PSS heterogeneity were examined in an attempt to improve the ability of baseline plaque features to stratify coronary plaque risk.

**Figure 5:**
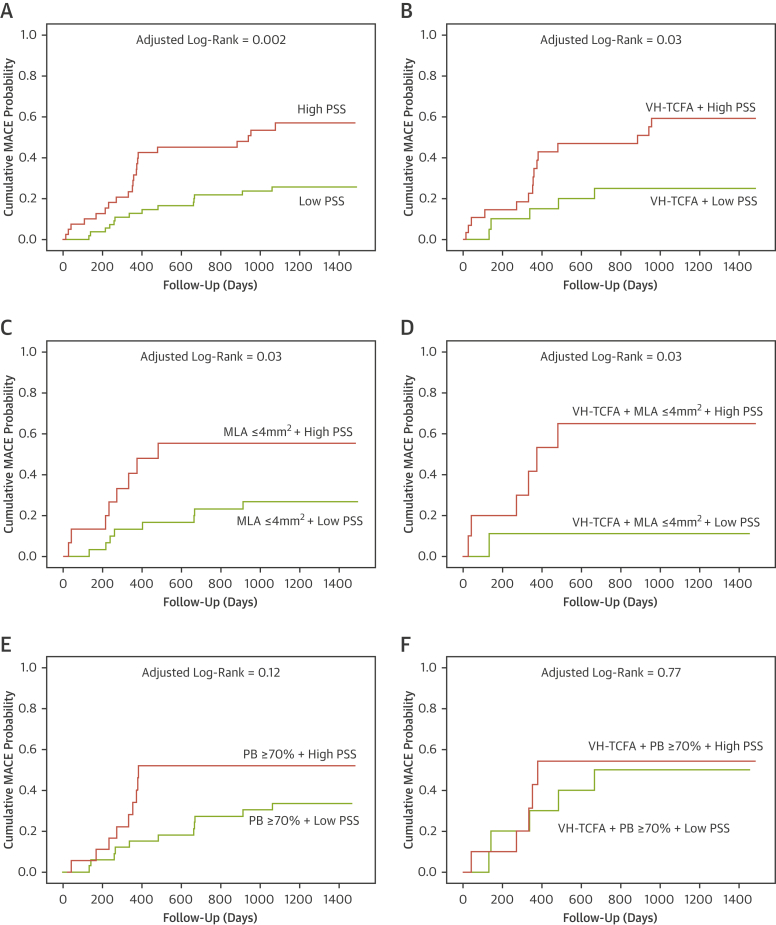
Time-to-Event Curves for MACE Rates According to Baseline Plaque Characteristics and PSS Group Cumulative MACE probability for **(A)** all plaques, **(B)** VH-TCFAs, **(C)** plaques with MLA ≤4 mm^2^, **(D)** VH-TCFA + MLA ≤4 mm^2^, **(E)** plaques with PB ≥70% at the MLA, and **(F)** VH-TCFA + PB ≥70% according to high or low PSS. All plaques = 101 (100%); VH-TCFA = 51 (50.5%); MLA ≤4 mm^2^ = 47 (46.5%); VH-TCFA + MLA ≤4 mm^2^ = 20 (19.8%); PB ≥70% at the MLA = 55 (54.5%); VH-TCFA + PB ≥70% = 21 (20.8%). Abbreviations as in Figures 1 and 3.

ROC analysis was used first to identify the PSS and heterogeneity in PSS cutoff thresholds that best predicted future MACE (Supplemental Figure 4). Including PSS in plaque assessment markedly improved the identification of nonculprit plaques that led to MACE for all plaques (p = 0.002) (Figure 5A), for VH-TCFAs (p = 0.03) (Figure 5B), for plaques with MLA ≤4 mm^2^ (p = 0.03) (Figure 5C), or for VH-TCFA plaques with an MLA ≤4 mm^2^ (p = 0.03) (Figure 5D) but not plaques with PB ≥70% at the MLA (p = 0.12) (Figure 5E) or VH-TCFA plaques with a PB ≥70% at the MLA (p = 0.77) (Figure 5F).

**Figure 6:**
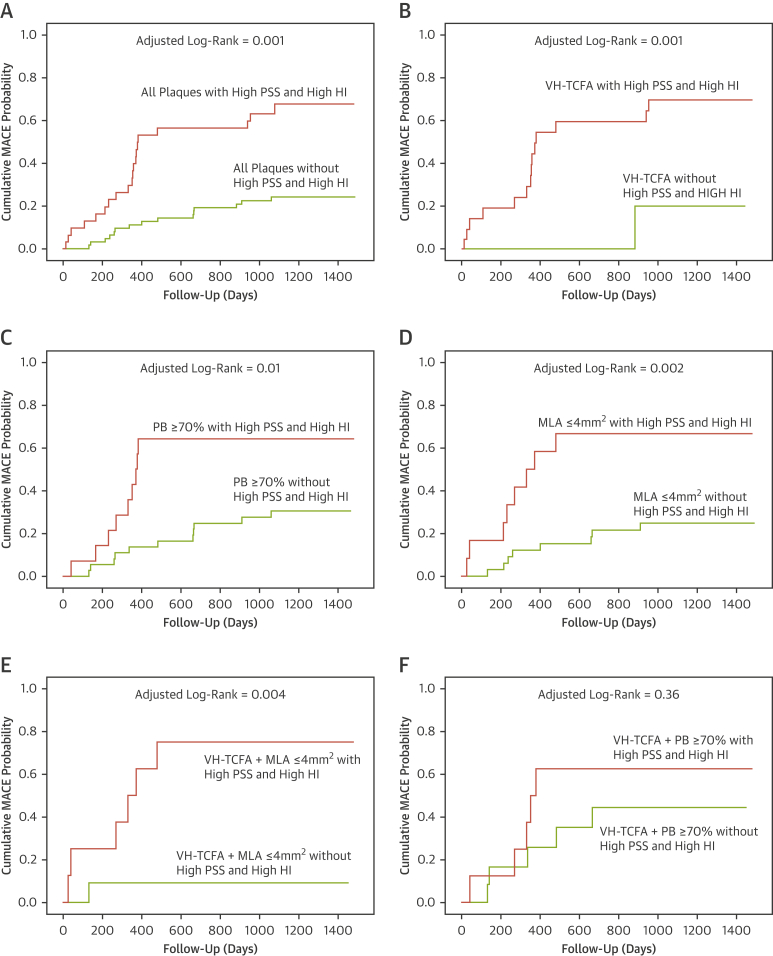
Time-to-Event Curves for MACE Rates According to Baseline Plaque Characteristics, PSS, and PSS Heterogeneity Index Groups Cumulative MACE probability for **(A)** all plaques, **(B)** VH-TCFAs, **(C)** plaques with PB ≥ 70% at the MLA, **(D)** plaques with MLA ≤4mm^2^, **(E)** VH-TCFA + MLA ≤4 mm^2^, and **(F)** VH-TCFA + PB ≥70% according to the presence or absence of high PSS or high HI. All plaques = 101 (100%); VH-TCFA = 51 (50.5%); MLA ≤4 mm^2^ = 47 (46.5%); VH-TCFA + MLA ≤4 mm^2^ = 20 (19.8%); PB ≥70% at the MLA = 55 (54.5%); VH-TCFA+PB≥70% = 21 (20.8%). HI = heterogeneity index; other abbreviations as in Figures 1 and 3.

Incorporating HI further improved the ability to use PSS as a means of distinguishing between MACE and no-MACE NCLs across a variety of plaque characteristics including all plaques (p = 0.001) (Figure 6A), VH-TCFA plaques (p = 0.001) (Figure 6B), PB ≥70% at the MLA site (p = 0.01) (Figure 6C), MLA ≤4 mm^2^ (p = 0.002) (Figure 6D), and VH-TCFA plaques with an MLA ≤4 mm^2^ (p = 0.004) (Figure 6E) but not VH-TCFA plaques with a PB ≥70% (p = 0.36) (Figure 6F). Further analysis demonstrated acceptable positive predictive values for high PSS and high HI across a variety of plaque subtypes (Supplemental Appendix).

## Discussion

Both post-mortem and prospective VH-IVUS studies have identified TCFA (or VH-TCFA) as the lesion most likely to rupture 2, 4, 5, 11. However, TCFAs are frequent in patients with coronary artery disease 2, 12; they are present in patients with both stable and unstable syndromes (13), and <10% actually lead to MACE within several years. Furthermore, MACE are not restricted to TCFAs, suggesting that factors additional to plaque morphology are important in determining which plaques act as precursors of future events.

PSS is regulated by plaque composition, geometry, luminal configuration, and hemodynamic factors (8), and may contribute to plaque risk stratification. In the VIVA study, PSS was increased in culprit plaques of patients who presented with ACS versus those with stable angina and in those that ruptured and led to future MACE 8, 9, 10. Furthermore, the combination of PSS estimates and IVUS provided results that were superior to those of IVUS alone in identifying plaques that led to MACE. However, VIVA was a small single-center study in 170 patients without angiographic confirmation of all MACE, and such findings require confirmation in larger, multicenter cohorts. It is also unclear whether there are PSS parameters that are better discriminators of MACE than those studied in VIVA.

The present study compared a cohort with NCL MACE from the 697-patient multicenter PROSPECT study with a propensity-matched control group (8). Although PSS did not differ between the 2 groups across the entire plaque length, significant differences were observed in the peri-MLA regions, sites where plaque destabilization is most likely to occur (14) (Central Illustration). More specifically, PSS and variations in PSS were higher in MACE plaques regardless of whether all plaques, VH-TCFAs, or plaques with MLA ≤4 mm^2^ were examined. Both parameters were also increased in NCL with PB ≥70% at the MLA, although this did not quite reach statistical significance; which is not unexpected because the propensity score matching process included only plaques with PB ≥60 at the MLA site.

**Central Illustration:**
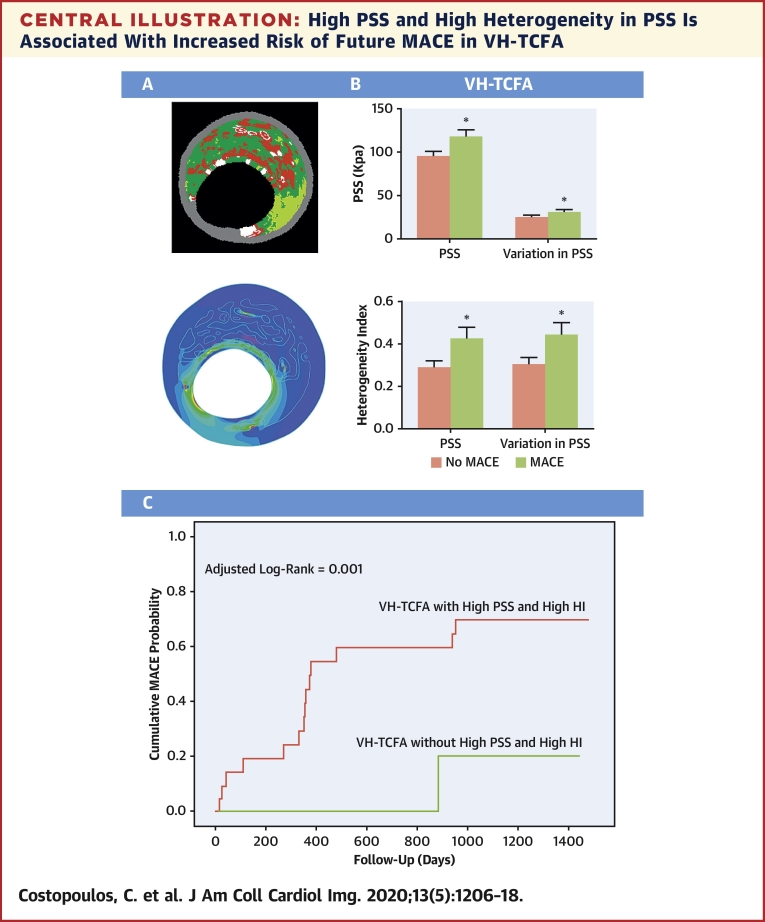
High PSS and High Heterogeneity in PSS Is Associated With Increased Risk of Future MACE in VH-TCFA **(A)** VH-IVUS and its associated band plot in a VH-TCFA. **(B)** PSS, variations in PSS and heterogeneity index of PSS is increased in VH-TCFAs with future MACE. **(C)** Incorporation of PSS and heterogeneity index in PSS allows the identification of VH-TCFAs that lead to MACE. *p < 0.05. HI = heterogeneity index; MACE = major adverse cardiovascular event(s); PSS = plaque structural stress; VH-TCFA = virtual-histology thin-cap fibroatheroma.

High PSS in the peri-MLA segments can lead to plaque destabilization either by triggering rupture or by increasing vulnerability through necrotic core growth. Increased variations in PSS between systole and diastole may also result in fibrous cap fatigue, further promoting plaque disruption. Heterogeneity in PSS and variations in PSS in peri-MLA segments were also increased in VH-TCFAs associated with MACE versus those in no-MACE patients (Central Illustration). This could be due to subtle differences in plaque composition or luminal geometry or both, which may result in points of fibrous cap weakness in the longitudinal direction (as opposed to the axial direction, hypothesized with increased variations in PSS). Increased PSS heterogeneity in conjunction with high PSS would therefore further promote plaque destabilization.

One aim of plaque imaging is to identify the subset of plaques at highest risk of MACE, so that therapy and monitoring can be adjusted according to prognosis. This is particularly important for new treatments such as PCSK9 inhibitor therapy (15) or canakinumab therapy (16), which may be too expensive for the entire population at risk. This study found that PSS and HI calculations significantly improved plaque risk stratification, an important consideration when most plaques classified as high-risk by imaging alone remain clinically silent (Central Illustration). Indeed, there was divergence of the time-to-event curves for both PSS and heterogeneity of PSS within 1 year of follow-up (Figures 4 and 5), which further implicates PSS in plaque destabilization. As PSS, like plaque morphology, can be highly dynamic over time, the effects of high PSS and/or PSS heterogeneity on plaque stability would be expected to occur closer to the time of high stress.

Similar to PSS, endothelial shear stress (ESS) has been shown recently to provide incremental risk stratification of untreated coronary lesions beyond IVUS plaque characterization (17). PSS describes the stress located inside an atherosclerotic plaque and is affected by vessel expansion and stretch by exposure to arterial pressure, whereas ESS describes the parallel friction force exerted by blood flow on the endothelial surface of the arterial wall. As PSS and ESS refer to different biomechanical forces, it is possible that the combination of ESS and PSS may improve plaque risk stratification further. Ultimately the combination of patient characteristics, plaque morphology, and biomechanical and inflammation plaque profiling may allow identification of those NCLs that will proceed to MACE, thus allowing more aggressive secondary prevention and also perhaps earlier definitive treatment.

### Study limitations

First, PSS calculations were applied retrospectively, and prospective studies should be performed to confirm the additive value of PSS and other PSS-related parameters in plaque risk assessment. However, propensity score matching ensured that a well-matched control group was used for comparison. Second, MACE in the PROSPECT study were largely driven by hospitalization for progressive and unstable angina, and although this could be due to plaque rupture with subsequent healing, other forms of plaque destabilization or plaque growth may be responsible. Third, PSS calculations based on VH-IVUS are limited by the resolution and ability of VH-IVUS to identify plaque components. However, IVUS is the only intravascular imaging modality to date with prospective clinical data. Finally, the role of ESS was not investigated, which can be important in a study where clinical events are likely driven by plaque growth. Combined PSS and ESS of the PROSPECT study would be of huge interest.

## Conclusions

PSS and variations in PSS in the peri-MLA regions were increased in nonculprit plaques, leading to MACE in the PROSPECT study across a variety of plaque subtypes. Longitudinal heterogeneity in PSS was also increased in plaques leading to MACE, especially VH-TCFAs. Incorporation of PSS and heterogeneity in PSS improves the ability of IVUS to predict MACE, suggesting that biomechanical modeling may have a role in coronary atherosclerotic plaque risk stratification.

Perspectives **COMPETENCY IN MEDICAL KNOWLEDGE:** PSS, variations in PSS and longitudinal heterogeneity in PSS is increased in the peri-MLA regions of plaques, including VH-TCFA, which proceed to future MACE. Incorporation of such biomechanical analysis improves the ability of IVUS to identify high-risk plaques.

**TRANSLATIONAL OUTLOOK:** PSS, which may be estimated from VH-IVUS images, has been proposed as a mechanism that determines rupture in high-risk regions. This study provides further evidence that PSS and other associated features may better identify plaques that cause future patient events, allowing earlier and more aggressive treatment of relevant risk factors.
