# Supplementary material for: Heterogeneity of Plaque Structural Stress Is Increased in Plaques Leading to MACE: Insights From the PROSPECT Study
Source: JACC Cardiovasc Imaging. 2020 May;13(5):1206–18. doi: 10.1016/j.jcmg.2019.05.024 (PMC7198978; doi:10.1016/j.jcmg.2019.05.024)
Supplement: Supplemental Data [file mmc1.docx]

**Supplement**

# Supplemental Methods

*Patient recruitment*

PROSPECT was a prospective study aiming to examine the natural history of atherosclerosis as assessed by angiography and IVUS. A total of 697 patients with ACS across 37 sites in the United States and Europe underwent 3-vessel greyscale and VH-IVUS after undergoing successful and uncomplicated PCI for the treatment of all lesions attributed to the index event. All patients recruited provided written informed consent.

Clinical inclusion criteria:

1. Age ≥ 18 years.

2. ACS (unstable angina, non ST-segment elevation myocardial infarction (NSTEMI) or ST-segment elevation myocardial infarction (STEMI) with ≥10 minutes of angina or anginal equivalent consistent with unstable angina or myocardial infarction (MI) within 72 hours, with either elevated cardiac biomarkers (CK-MB or troponin) or ST-segment deviation of >1 mm in ≥2 contiguous electrocardiographic leads.

3. Patient agrees and is able to follow all protocol procedures.

4. Patient provides written, informed consent.

Angiographic inclusion criteria were:

1. Successful and uncomplicated PCI performed in one or two major epicardial coronary arteries (including their branches). Successful PCI was defined as residual diameter stenosis of < 50% in all lesions with TIMI-3 flow in all vessels. Uncomplicated PCI was defined as the absence of intra-procedural chest pain or ST-segment changes lasting > 10 minutes, sustained vessel closure, slow or no re-flow, side branch loss, distal embolization, perforation, residual dissection, or requirement for cardiopulmonary resuscitation, cardioversion or defibrillation, pacemaker insertion, intubation, intra-aortic balloon insertion or intravenous pressors.

Clinical exclusion criteria:

1. STEMI within 24 hours.
2. Serum creatinine ≥2.5 mg/dl.
3. Decompensated hypotension, heart failure, shock, refractory ventricular arrhythmias, acute conduction system disease, implanted defibrillator, or left ventricular ejection fraction ≤ 30%.
4. Known severe allergy, hypersensitivity or contraindication to aspirin, heparin, thienopyridines or contrast which cannot be adequately pre-medicated.
5. Stroke or transient ischemic attack within 6 months.
6. Significant gastrointestinal or urinary bleed within the past 6 months, coagulopathy, bleeding diathesis, or refusal of blood transfusions.
7. Percutaneous coronary intervention (PCI) within 6 months or any prior bypass graft surgery.
8. Prior or planned heart transplant or any other organ transplant.
9. Anticipated life expectancy < 1 year.
10. Prior participation in the study or current enrolment in another investigational study that has not reached its primary endpoint.
11. Pregnant or nursing patients and those who plan pregnancy in the period up to one year following index procedure. Female patients of child-bearing potential must have had a negative pregnancy test done within 7 days prior to the index procedure.

Angiographic exclusion criteria were:

1. PCI required in all 3 major epicardial coronary arteries.

2. Anatomy not suitable for 3-vessel IVUS.

3. Left main coronary artery culprit lesion.

4. Any remaining lesion with diameter stenosis > 50% after PCI.

5. Coronary artery bypass graft surgery planned within 1-year after PCI.

VH-IVUS was performed in all main coronary arteries (6 to 8cm of the proximal segments) after the administration of intra-coronary glyceryl trinitrate. Data were acquired with 20MHz Eagle-Eye Gold catheters (Volcano Corporation, Rancho Cordova, California, USA) using motorized pullback at 0.5mm/s. Radiofrequency data were gathered in diastole at each R-wave peak by patient-specific ECG-gating. A total of 2811 plaques were identified the analysis of which was performed offline by the Cardiovascular Research Foundation core laboratory, using the Volcano Image Analysis Software.

Independent study clinicians verified all major adverse cardiovascular events (MACE) defined as the composite of death from cardiac causes, cardiac arrest, myocardial infarction, or rehospitalization due to unstable or progressive angina according to the Braunwald Unstable Angina Classification and the Canadian Cardiovascular Society Angina Classification). An independent review panel adjudicated each suspected MACE and on the basis of follow-up angiography, MACE were adjudicated to culprit lesions or at previously untreated coronary segments (non-culprit lesions). In the cases where follow-up angiography was not available, the site associated with the event was classified as indeterminate. In patients with multiple non-culprit MACE the first event was chosen.

*Biomechanical modeling*

Plaque geometry was constructed from VH-IVUS data using an in-house MATLAB code (D3Plaque, Cambridge, UK). Any frames where PB < 40% were excluded from biomechanical analysis, as these were considered non-atherosclerotic regions. As *in vivo* data are recorded during diastole, circumferential shrinkage was applied to generate a zero-pressure condition for computational simulation as previously described^1, 2^. Plaque components were assumed to be incompressible, piecewise homogeneous, non-linear isotropic and hyper-elastic as described by the modified Mooney-Rivlin strain energy density function:

$$W=c_{1}\left( \bar{I}_{1}-3 \right)+D_{1}\left[ e^{D_{2}\left( \bar{I}_{1}-3 \right)}-1 \right]+\kappa\left( J-1 \right)$$

where $\bar{I}_{1}=J^{-2/3}I_{1}$ with $I_{1}$ being the first invariant of the unimodular component of the left Cauchy-Green deformation tensor. $J=det\left( \boldsymbol{F} \right)$ and ***F*** is the deformation gradient. $\kappa$ is the Lagrangian multiplier for the incompressibility. $c_{1}$, $D_{1}$ and $D_{2}$ are material parameters derived from previous experimental work^3^ and include; arterial vessel wall, *c*_1_=0.138 kPa, *D*_1_=3.833 kPa, *D*_2_=18.803; fibrous tissue, *c*_1_=0.186 kPa, *D*_1_=5.769 kPa, *D*_2_=18.219 and necrotic core, *c*_1_=0.046 kPa, *D*_1_=4.885 kPa, *D*_2_=5.426. The material properties of dense calcification were derived by fitting a Young’s modulus of 184 MPa derived from experimental work^4^: *c*_1_=1.147×10^5^ kPa, *D*_1_=7.673×10^4^ kPa and *D*_2_=2.838×10^-8^. The motion of each atherosclerotic component is governed by kinetic equations as:

$$\rho v_{i,tt}=\sigma_{ij,j} (i, j=1, 2)$$

where $[v_{i}]$ and $[\sigma_{ij}]$ are the displacement vector and stress tensor, respectively, $\rho$ is the density of each component and $t$ stands for time.

The entire plaque geometric model was meshed using 9-node quadrilaterals (generating approximately 10,000 elements and 40,000 nodes per model). Displacement and strain were assumed to be large. There was no relative movement at the interface of atherosclerotic components and the relative energy tolerance was set to be 0.005. Two adjacent points were fixed to prevent rigid body displacement. PSS was used to characterize the mechanical loading within the plaque structure in the peri-luminal region (0.2mm maximum depth from the luminal contour). Dynamic loading conditions were generated from coronary pressure recordings taken at the time of the procedure. Pressure at the outer boundary was set to zero. All simulations were performed using ADINA 8.6.1 (ADINA R&D, Inc., USA) software.

*Statistical Analysis*

The method of propensity score matching was as follows:

1. Only lesions with plaque burden ≥ 60% were included.

2. NCLs with MACE : NCLs with no MACE =1:2.

3. One lesion per patient.

4. If a patient had multiple NCL MACE and multiple NCLs with plaque burden≥60%, the lesion associated with the first MACE was chosen.

5. If a patient had multiple NCLs with PB ≥ 60%, the lesion with the greatest PB was chosen.

6. Patients with NCL MACE related to NCL that were not identified at baseline or without valid VH-IVUS data were eliminated from the analysis.

7. Propensity matching parameters:

1. Patient level parameters: Insulin treated DM, prior PCI, age (continuous) and gender.
2. Lesion level parameters: Plaque burden at MLA (minimum lumen area), MLA, plaque phenotype (TCFA, ThCFA, other)
3. Plaque burden at MLA and MLA was used as continuous variable.

Multiple imputations were performed on the matching variables to avoid losing lesions/patients when running the logistic regression on subjects with missing values for some of the variables. The propensity score (per patient) was calculated as an average of the propensity scores from 10 imputed datasets.

The 2:1 matching was performed using a caliper of 0.5 (0.2 multiplied by the logit of the standard deviation of the propensity score).^5^ Important patient demographics and VH-IVUS characteristics before and after propensity score matching are demonstrated in **Supplemental Table 1**.

**Supplemental Results**

*Positive predictive value of high PSS and high HI*

The positive predictive value of high PSS and high HI in identifying lesions that proceed to MACE was calculated for the entire plaque population as well all plaque subtypes described in **Figure 6** (all plaques=60.6%, VH-TCFA=63.6%, PB ≥ 70%=56.3%, MLA ≤4mm^2^=66.7%, VH-TCFA+MLA ≤4mm^2^ =75% VH-TCFA+PB ≥ 70% = 55.6%).

Supplemental References

1. Tang D, Teng Z, Canton G, Hatsukami TS, Dong L, Huang X and Yuan C. Local critical stress correlates better than global maximum stress with plaque morphological features linked to atherosclerotic plaque vulnerability: an in vivo multi-patient study. *Biomed Eng Online*. 2009;8:15.

2. Huang Y, Teng Z, Sadat U, Hilborne S, Young VE, Graves MJ and Gillard JH. Non-uniform shrinkage for obtaining computational start shape for in-vivo MRI-based plaque vulnerability assessment. *J Biomech*. 2011;44:2316-9.

3. Teng Z, Zhang Y, Huang Y, Feng J, Yuan J, Lu Q, Sutcliffe MP, Brown AJ, Jing Z and Gillard JH. Material properties of components in human carotid atherosclerotic plaques: a uniaxial extension study. *Acta Biomater*. 2014;10:5055-63.

4. Ebenstein DM, Coughlin D, Chapman J, Li C and Pruitt LA. Nanomechanical properties of calcification, fibrous tissue, and hematoma from atherosclerotic plaques. *J Biomed Mater Res A*. 2009;91:1028-37.

5. Austin PC. Optimal caliper widths for propensity-score matching when estimating differences in means and differences in proportions in observational studies. *Pharm Stat*. 2011;10:150-61.

**Supplemental Table 1.** Patient demographics and VH-IVUS characteristics before and after propensity score matching

|  | **Before Propensity Score Matching** | | | **After Propensity Score Matching** | | |
| --- | --- | --- | --- | --- | --- | --- |
| **Characteristic** | **MACE (n=35)** | **No MACE (n=384)** | **p-value** | **MACE (n=35)** | **No MACE (n=66)** | **p-value** |
| **Patient characteristics** |  |  |  |  |  |  |
| Age, years | 54.9 [49.1 - 65.7] | 58.8 [51.4 - 67.4 ] | 0.21 | 54.9 [49.1 - 65.7] | 58.8 [50.1 - 66.4] | 0.42 |
| Male | 29/35 (82.9) | 202/384(52.6) | 0.27 | 29/35 (82.9) | 53/66 (80.3) | 0.75 |
| Diabetes | 10/35 (28.6) | 60/383 (15.7) | 0.05 | 10/35 (28.6) | 13/66 (19.7) | 0.31 |
| Body Mass Index (kg/m^2^) | 29.8 [25.3 - 32.9] | 27.8 [ 25.1 - 31.1] | 0.13 | 29.8 [25.3 - 32.9] | 27.7 [24.9 - 30.8] | 0.15 |
| Prior Myocardial Infarction | 4/35 (11.4) | 42/383 (11.0) | 1.00 | 4/35 (11.4) | 9/66 (13.6) | 1.00 |
| CAD (stenosis≥50%) | 6/32 (18.8) | 48/377 (12.7) | 0.41 | 6/32 (18.8) | 13/65 (20.0) | 0.88 |
| Previous PCI | 5/35 (14.3) | 39/383 (10.2) | 0.40 | 5/35 (14.3) | 9/66 (13.6) | 1.00 |
| Family History of CAD | 13/28 (46.4) | 150/338 (44.4) | 0.83 | 13/28 (46.4) | 25/57 (43.9) | 0.82 |
| Hypertension Requiring Medication | 19/33 (57.6) | 181/383 (47.3) | 0.26 | 19/33 (57.6) | 34/65 (52.3) | 0.62 |
| Hypercholesterolemia | 15/28 (53.6) | 162/360 (45.0) | 0.38 | 15/28 (53.6) | 32/65 (49.2) | 0.70 |
| History of Tobacco Use | 19/35 (54.3) | 186 /379 (49.1) | 0.56 | 19/35 (54.3) | 29/66 (43.9) | 0.32 |
| Clinical Presentation (STEMI) | 10/35 (28.6) | 113/384(29.4) | 0.92 | 10/35 (28.6) | 13/66 (19.7) | 0.31 |
| Clinical Presentation (NSTEMI) | 24/35 (68.6) | 255/384 (66.4) | 0.79 | 24/35 (68.6) | 48/66 (72.7) | 0.66 |
| Clinical Presentation (UA) | 1/35 (2.9) | 16/384 (4.2) | 1.00 | 1/35 (2.9) | 5/66 (7.6) | 0.66 |
| Tot. Cholesterol/HDL-Cholesterol | 4.6 [3.5 - 5.5] | 4.3 [3.4 - 5.3] | 0.27 | 4.6 [3.5 - 5.5] | 4.3 [3.2 - 5.0] | 0.26 |
| Metabolic Syndrome | 16/34 (47.1) | 176/372 (47.3) | 0.98 | 16/34 (47.1) | 29/63 (46.0) | 0.92 |
| **VH-IVUS characteristics** |  |  |  |  |  |  |
| *Lesion Phenotype* |  |  |  |  |  |  |
| VH-TCFA | 20/35 (57.1) | 121/384 (31.5) | 0.002 | 20/35 (57.1) | 31/66 (47.0) | 0.33 |
| VH-ThCFA | 12/35 (34.3) | 169/384 (44.0) | 0.27 | 12/35 (34.3) | 26/66 (39.4) | 0.61 |
| VH-PIT | 2/35 (5.7) | 85/384 (22.1) | 0.02 | 2/35 (5.7) | 9/66 (13.6) | 0.32 |
| VH-FCa | 1/35 (2.9) | 5/384 (1.3) | 0.41 | 1/35 (2.9) | 0/66 (0.0) | 0.35 |
| VH-FT | 0/35 (0.0) | 4/384 (1.0) | 1.0 | 0/35 (0.0) | 0/66 (0.0) | NA |
| VH-TCFA or VH-ThCFA | 32/35 (91.4) | 290/384 (75.5) | 0.03 | 32/35 (91.4) | 57/66 (86.4) | 0.54 |
| *Plaque Data* |  |  |  |  |  |  |
| % NC Volume | 15.5 [9.0 - 21.5] | 13.0 [7.4 - 19.3] | 0.30 | 15.5 [9.0 - 21.5] | 14.9 [8.8 - 23.1] | 0.73 |
| % DC Volume | 6.1 [2.7 - 9.8] | 5.3 [2.4 - 9.1] | 0.47 | 6.1 [2.7 - 9.8] | 6.3 [3.6 - 9.4] | 0.95 |
| % FT Volume | 60.7 [54.5 - 65.9] | 59.1 [54.0 - 64.0] | 0.53 | 60.7 [54.5 - 65.9] | 57.6 [53.7 - 62.0] | 0.22 |
| % FF Volume | 15.2 [10.9 - 22.5] | 18.4 [12.2 - 26.1] | 0.15 | 15.2 [10.9 - 22.5] | 16.4 [10.2 - 23.2] | 0.78 |
| *MLA Site Data* |  |  |  |  |  |  |
| % NC CSA (%) | 13.9 [8.7 - 25.1] | 14.3 [6.5 - 22.7] | 0.32 | 13.9 [8.7 - 25.1] | 16.6 [8.9 - 29.1] | 0.44 |
| % DC CSA (%) | 5.0 [2.3 - 10.9] | 4.2 [1.4 - 9.4] | 0.50 | 5.0 [2.3 - 10.9] | 6.0 [2.0 - 11.4] | 0.73 |
| % FT CSA (%) | 61.1 [53.7 - 68.8] | 60.5 [51.3 - 66.4] | 0.31 | 61.1 [53.7 - 68.8] | 58.7 [49.0 - 64.2] | 0.11 |
| % FF CSA (%) | 11.1 [7.0 - 22.0] | 16.1 [8.6 - 26.4] | 0.11 | 11.1 [7.0 - 22.0] | 12.6 [5.8 - 22.2] | 0.80 |
| *Data present as median (interquartile range) or n (%).*  *CAD=coronary artery disease; HDL=high-density lipoprotein; LDL=low-density lipoprotein; NSTEMI=non-ST segment-elevation myocardial infarction; STEMI= ST segment-elevation myocardial infarction; UA=unstable angina* | | | | | | |

**Supplemental Table 2.** Drug therapy on discharge and over follow-up

| **Medications** | **MACE (n=35)** | **No MACE (n=66)** | **p-value** |
| --- | --- | --- | --- |
| **Cardiac Medications** |  |  |  |
| Statins: Discharge | 80.0% (28/35) | 87.9% (58/66) | 0.29 |
| Statins: 1095-Days | 78.1% (25/32) | 83.6% (46/55) | 0.52 |
|  |  |  |  |
| Lipid Lowering (not Statin): Discharge | 20.0% (7/35) | 9.1% (6/66) | 0.13 |
| Lipid Lowering (not Statin): 1095-Days | 31.3% (10/32) | 10.9% (6/55) | 0.02 |
|  |  |  |  |
| Any Lipid Lowering: Discharge | 91.4% (32/35) | 89.4% (59/66) | 1.00 |
| Any Lipid Lowering: 1095-Days | 90.6% (29/32) | 85.5% (47/55) | 0.74 |
|  |  |  |  |
| Aspirin: Discharge | 97.1% (34/35) | 92.4% (61/66) | 0.66 |
| Aspirin: 1095-Days | 93.8% (30/32) | 89.1% (49/55) | 0.70 |
|  |  |  |  |
| Clopidogrel: Discharge | 97.1% (34/35) | 95.5% (63/66) | 1.00 |
| Clopidogrel: 1095-Days | 40.6% (13/32) | 40.0% (22/55) | 0.95 |
|  |  |  |  |
| Ticlopidine: Discharge | 2.9% (1/35) | 4.5% (3/66) | 1.00 |
|  |  |  |  |
| Thienopyridines: Discharge | 97.1% (34/35) | 97.0% (64/66) | 1.00 |
| Thienopyridines: 1095-Days | 40.6% (13/32) | 40.0% (22/55) | 0.95 |
|  |  |  |  |
| Coumadin: Discharge | 0.0% (0/35) | 1.5% (1/66) | 1.00 |
| Coumadin: 365-Days | 0.0% (0/35) | 1.8% (1/57) | 1.00 |
|  |  |  |  |
| ACE Inhibitors: Discharge | 60.0% (21/35) | 68.2% (45/66) | 0.41 |
| ACE Inhibitors: 1095-Days | 46.9% (15/32) | 61.8% (34/55) | 0.18 |
|  |  |  |  |
| ARB: Discharge | 5.7% (2/35) | 12.3% (8/65) | 0.49 |
| ARB: 1095-Days | 15.6% (5/32) | 23.6% (13/55) | 0.37 |
|  |  |  |  |
| Beta Blockers: Discharge | 85.7% (30/35) | 92.4% (61/66) | 0.31 |
| Beta Blockers: 1095-Days | 84.4% (27/32) | 76.4% (42/55) | 0.37 |
|  |  |  |  |
| **Non-Cardiac Medications** |  |  |  |
| Diabetic Medication: Discharge | 25.7% (9/35) | 16.7% (11/66) | 0.28 |
| Diabetic Medication: 1095-Days | 25.0% (8/32) | 16.4% (9/55) | 0.33 |
|  |  |  |  |
| HRT: Discharge | 2.9% (1/35) | 4.5% (3/66) | 1.00 |
| HRT: 1095-Days | 3.1% (1/32) | 1.8% (1/55) | 1.00 |
| *Data are presented as % (n)*  *ACE=angiotensin-converting enzyme; ARB=angiotensin II receptor blocker; HRT=hormone replacement therapy* | | | |

**Supplemental Table 3.** Patient quantitative coronary analysis

| **Characteristic** | **MACE (n=35)** | **No MACE (n=66)** | **p-value** |
| --- | --- | --- | --- |
| Total length of coronary tree, mm | 411.0 [375.0, 498.0] | 437.2 [400.5, 484.5] | 0.42 |
| Average length of NCL, mm | 37.1 [16.7, 53.4] | 23.6 [10.4, 48.2] | 0.12 |
| Any lesions with DS% ≥50% | 45.7% (16/35) | 52.6% (30/57) | 0.52 |
| **Number of Diseased Vessels** |  |  |  |
| No. with 1 vessel CAD (DS>30%) | 8.6% (3/35) | 18.2% (12/66) | 0.20 |
| Number with 2 vessel CAD | 40.0% (14/35) | 37.9% (25/66) | 0.83 |
| Number with 3 vessel CAD | 51.4% (18/35) | 43.9% (29/66) | 0.47 |
| **Number of Vessels with lesions** |  |  |  |
| Number of vessels with lesions | 2.0 [1.0, 3.0] | 2.0 [1.0, 2.0] | 0.27 |
| Number of vessels with lesions: 0 | 8.6% (3/35) | 16.7% (11/66) | 0.37 |
| Number of vessels with lesions: 1 | 25.7% (9/35) | 24.2% (16/66) | 0.87 |
| Number of vessels with lesions: 2 | 37.1% (13/35) | 39.4% (26/66) | 0.82 |
| Number of vessels with lesions: 3 | 28.6% (10/35) | 19.7% (13/66) | 0.31 |
| **Number of lesions** |  |  |  |
| Number of lesions | 4.0 [3.0, 5.0] | 3.0 [1.0, 4.0] | 0.01 |
| Side branch lesions with DS>50% | 5.7% (2/35) | 3.0% (2/66) | 0.61 |
| Side branch lesion with DS>75% | 0.0% (0/35) | 1.5% (1/66) | 1.00 |
| *Data are present as median (interquartile range) or % (n)*  *CAD=coronary artery disease; DS=diameter stenosis; NCL=non-culprit lesions PCI=percutaneous coronary intervention; QCA=quantitative coronary analysis* | | | |

Supplemental Table 4. Grayscale IVUS non-culprit lesion characteristics

| **Characteristic** | **MACE (n=35)** | **No MACE (n=66)** | **p-value** |
| --- | --- | --- | --- |
| **Lesion Data** |  |  |  |
| Lesion Length (mm) | 30.4 [19.5 - 41.4] | 24.3 [15.7 - 36.7] | 0.11 |
| Total EEM Volume (mm^3^) | 474.1 [286.3 - 684.7] | 373.0 [183.4 - 602.3] | 0.09 |
| Total Lumen Volume (mm^3^) | 191.1 [130.0 - 288.5] | 165.9 [85.6 - 249.2] | 0.10 |
| Plaque Volume (%) | 55.0 [51.7 - 60.0] | 55.0 [52.0 - 59.7] | 0.86 |
| Av. EEM CSA (mm^3^/mm) | 15.4 [12.1 - 20.1] | 15.6 [12.7 - 18.2] | 0.61 |
| Av. Lumen CSA (mm^3^/mm) | 7.0 [5.4 - 8.4] | 6.7 [5.7 - 8.1] | 0.97 |
| Av. Plaque + Media CSA (mm^3^/mm) | 8.2 [6.8 - 11.6] | 8.4 [7.1 - 10.6] |  |
| **Morphology Data** |  |  |  |
| Echolucent Plaque | 12/35 (34.3) | 8/66 (12.1) | 0.008 |
| Plaque Rupture | 2/35 (5.7) | 4/66 (6.1) | 1.00 |
| **MLA Site Data** |  |  |  |
| EEM CSA (mm^2^) | 13.5 [10.9 - 17.5] | 14.9 [11.3 - 17.6] | 0.81 |
| MLA (mm^2^) | 4.1 [3.2 - 4.9] | 4.0 [3.4 - 5.1] | 0.62 |
| MLA≤4mm^2^ | 16/35 (45.7) | 31/66 (47.0) | 0.90 |
| Plaque & Media CSA (mm^2^) | 9.6 [7.7 - 12.2] | 10.4 [7.8 - 12.4] | 0.96 |
| Plaque Burden (%) | 70.1 [68.6 - 74.8] | 70.5 [66.9 - 73.3] | 0.35 |
| Plaque Burden ≥70% | (20/35) (57.1) | 35/66 (53.0) | 0.69 |
| Min. Lumen Diameter (mm) | 2.09 [1.91 - 2.20] | 2.10 [1.91 - 2.27] | 0.38 |
| Remodeling Index | 0.88 [0.81 - 1.01] | 0.95 [0.85 - 1.04] | 0.17 |
| *Data are present as median (interquartile range) or n (%).*  *CSA=cross-sectional area; EEM=external elastic membrane; MLA=minimum luminal area* | | | |


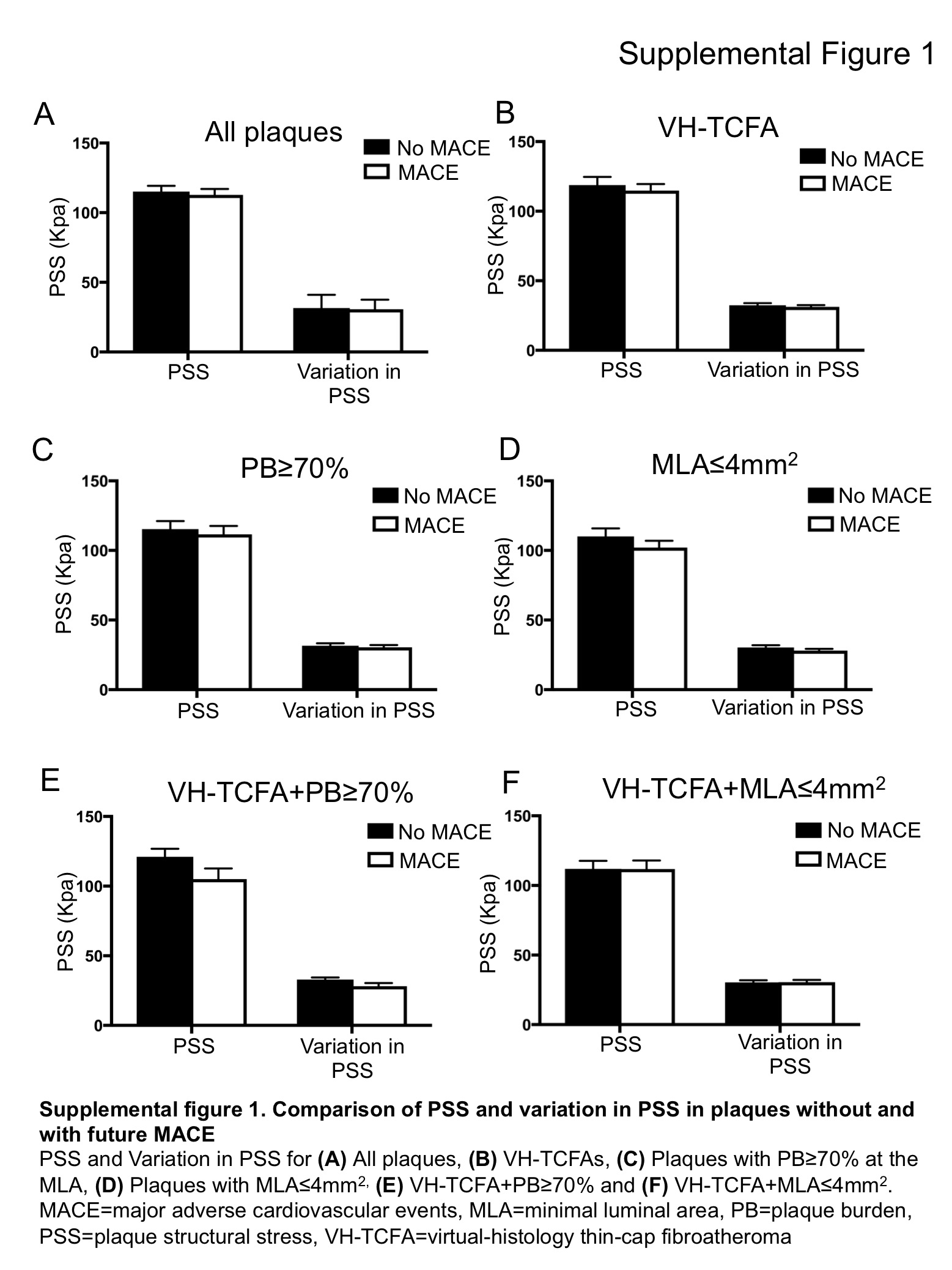


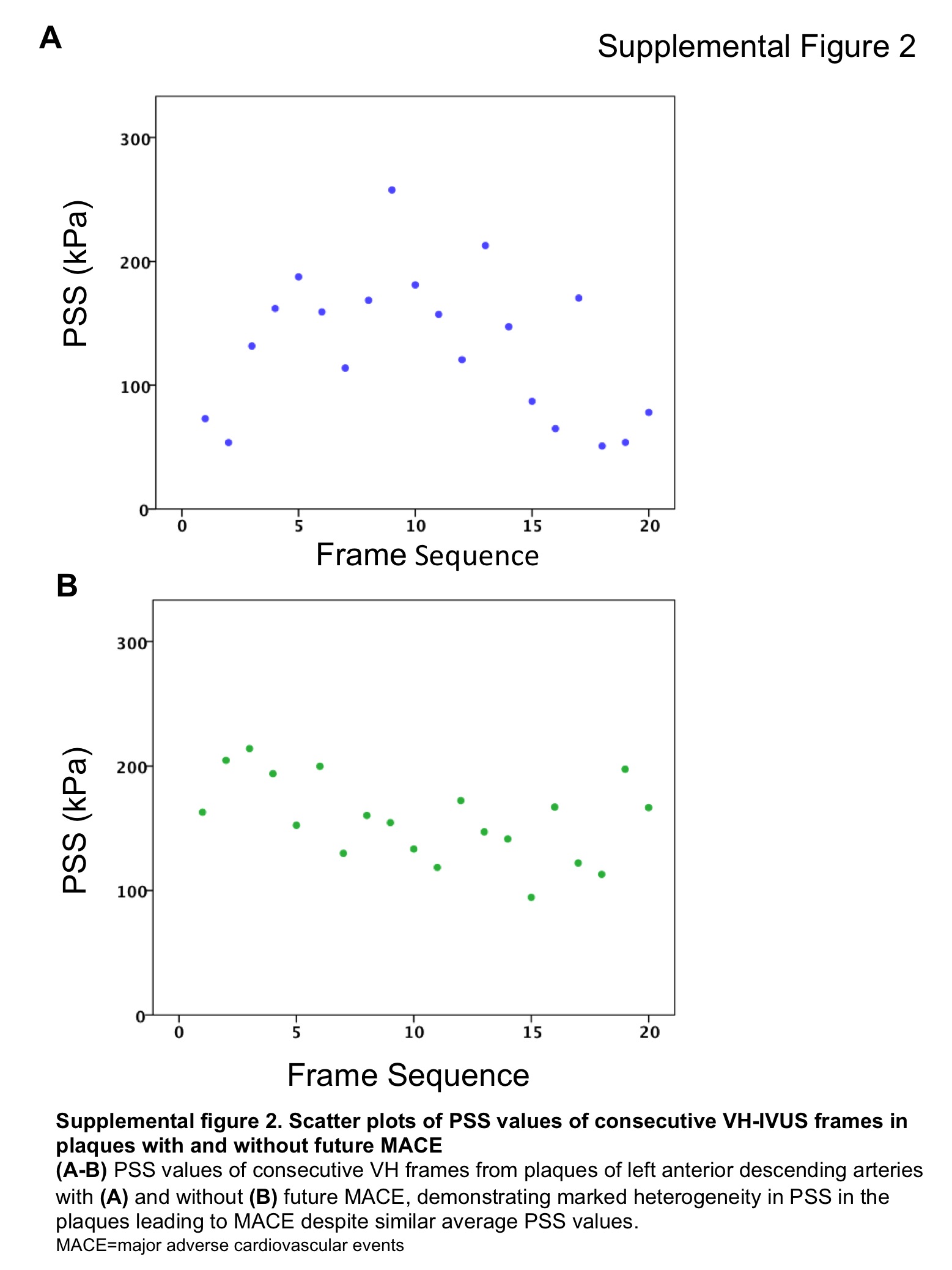


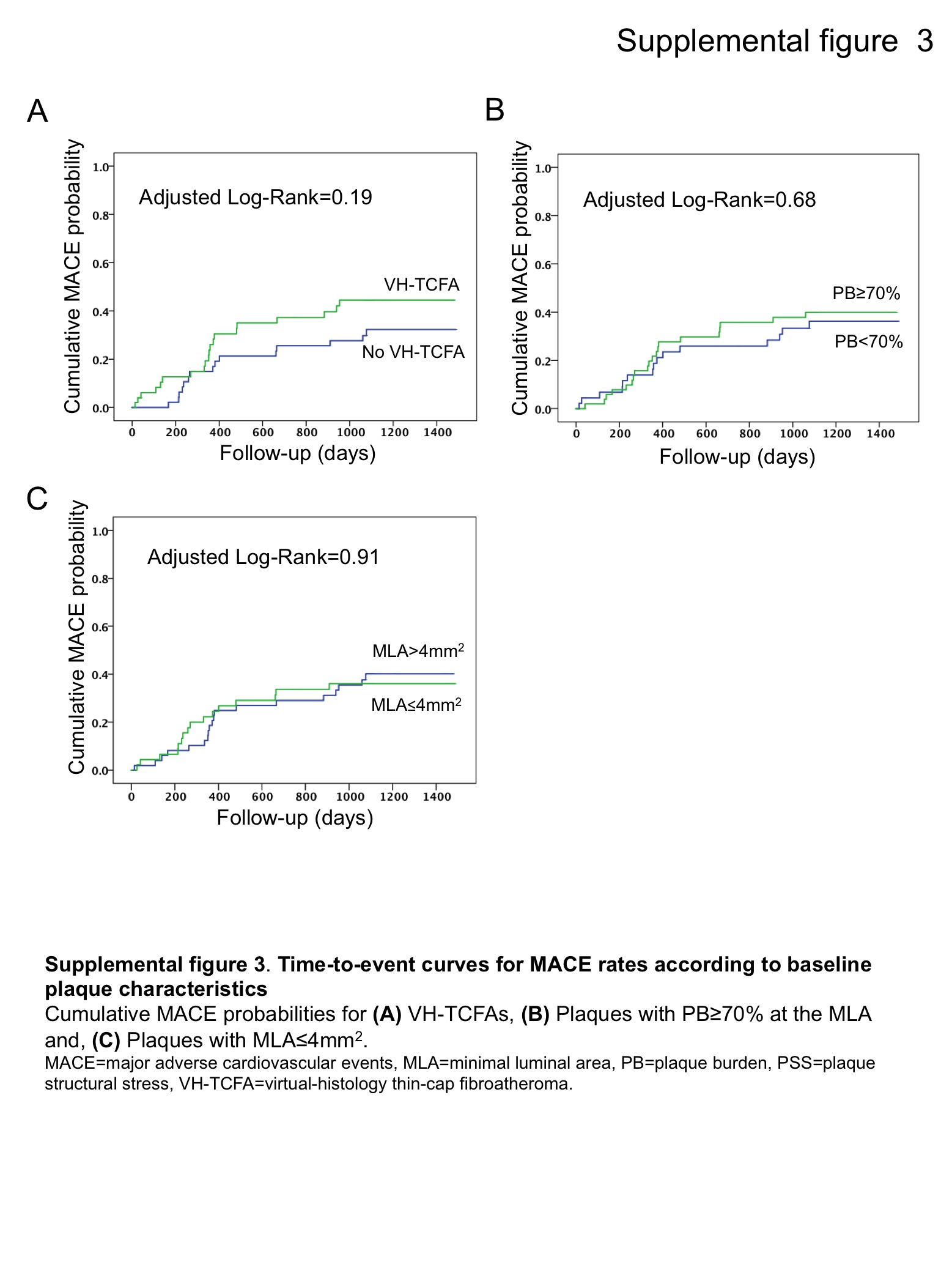


**
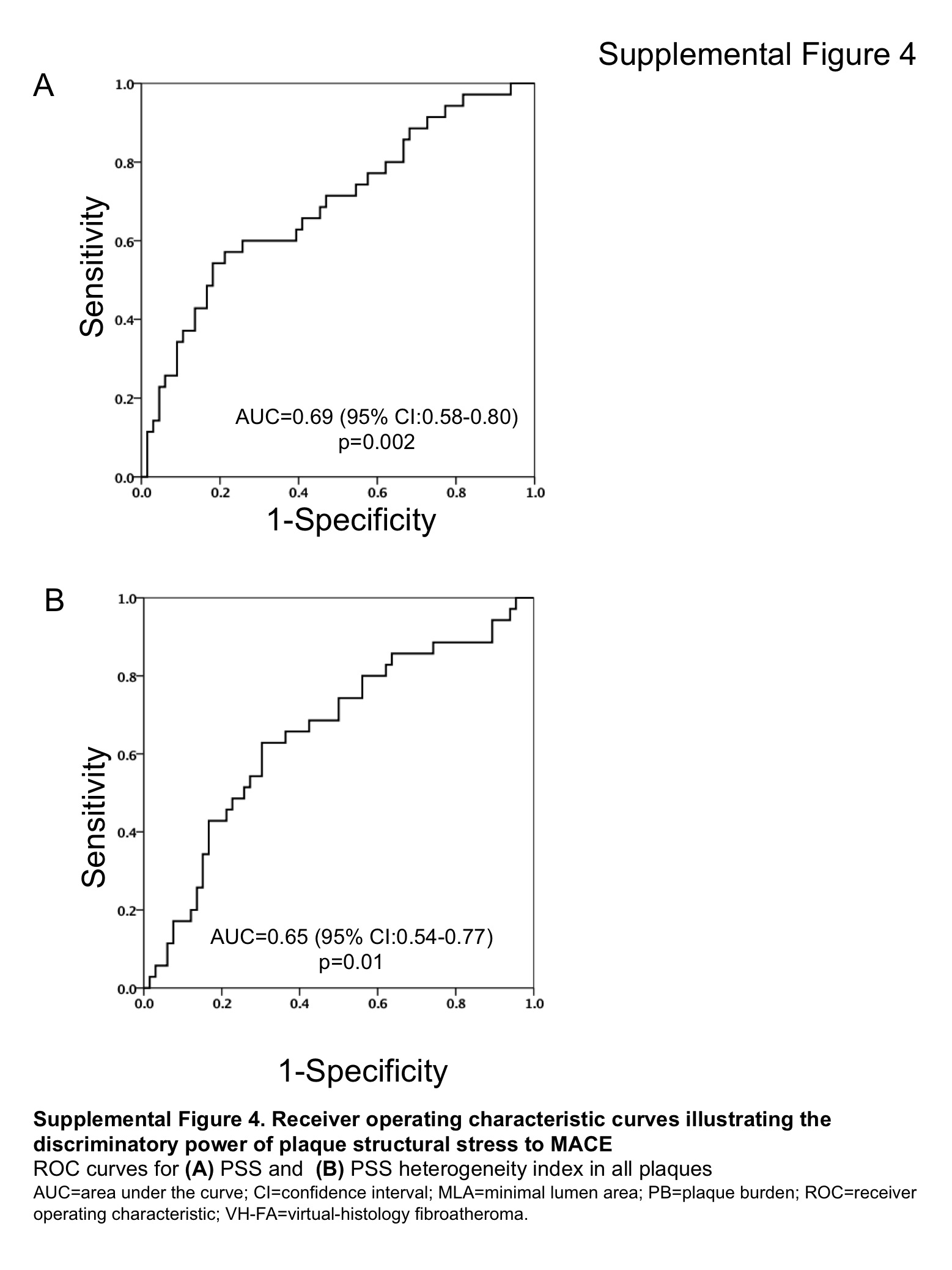
**
